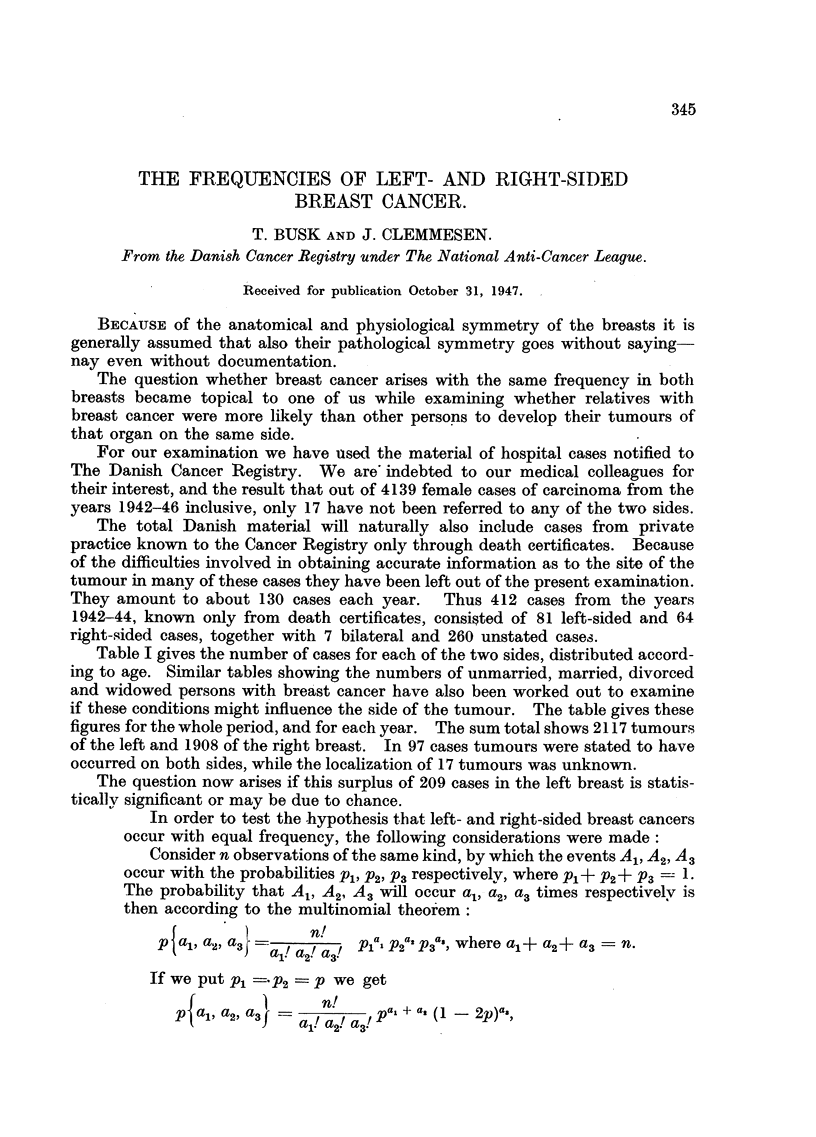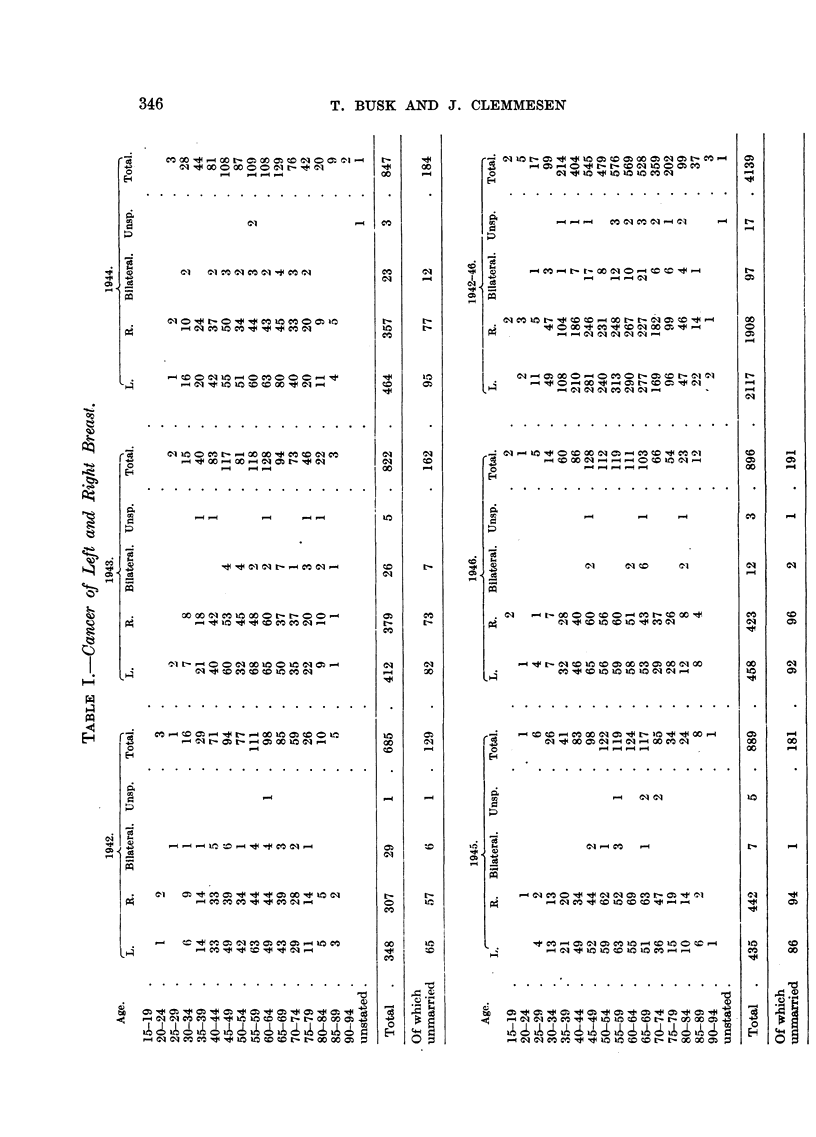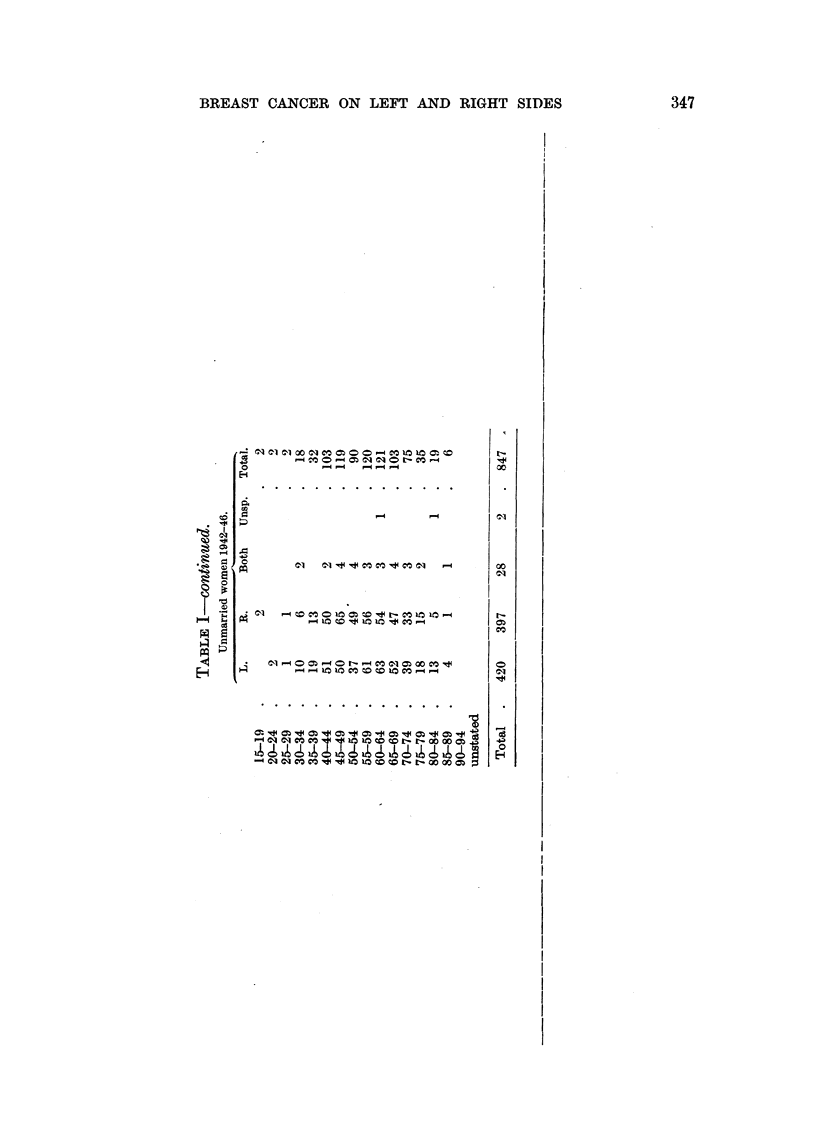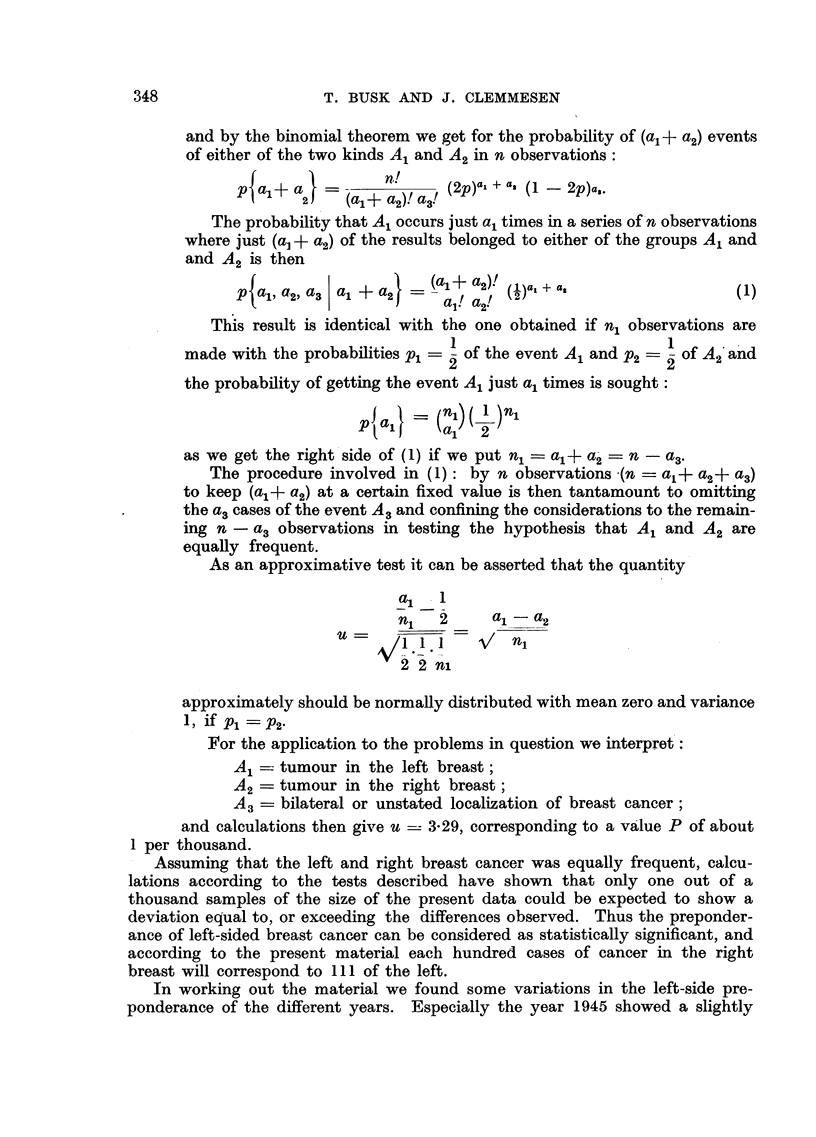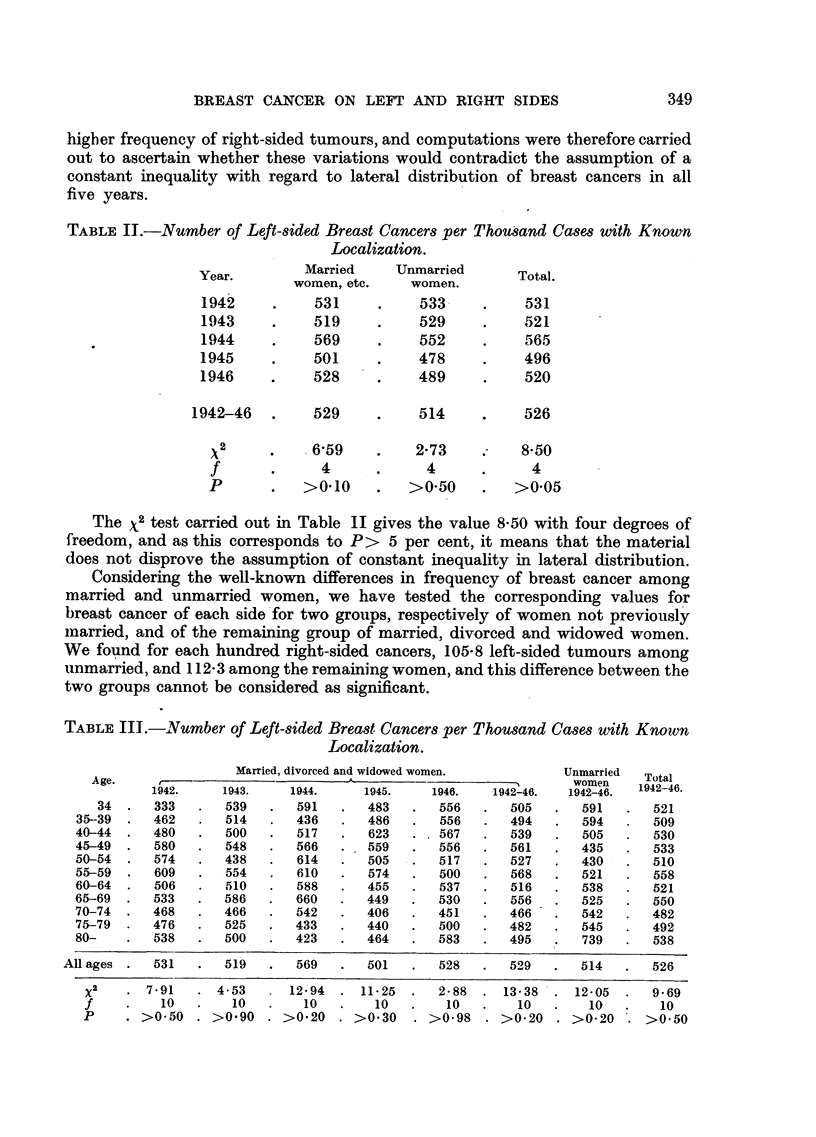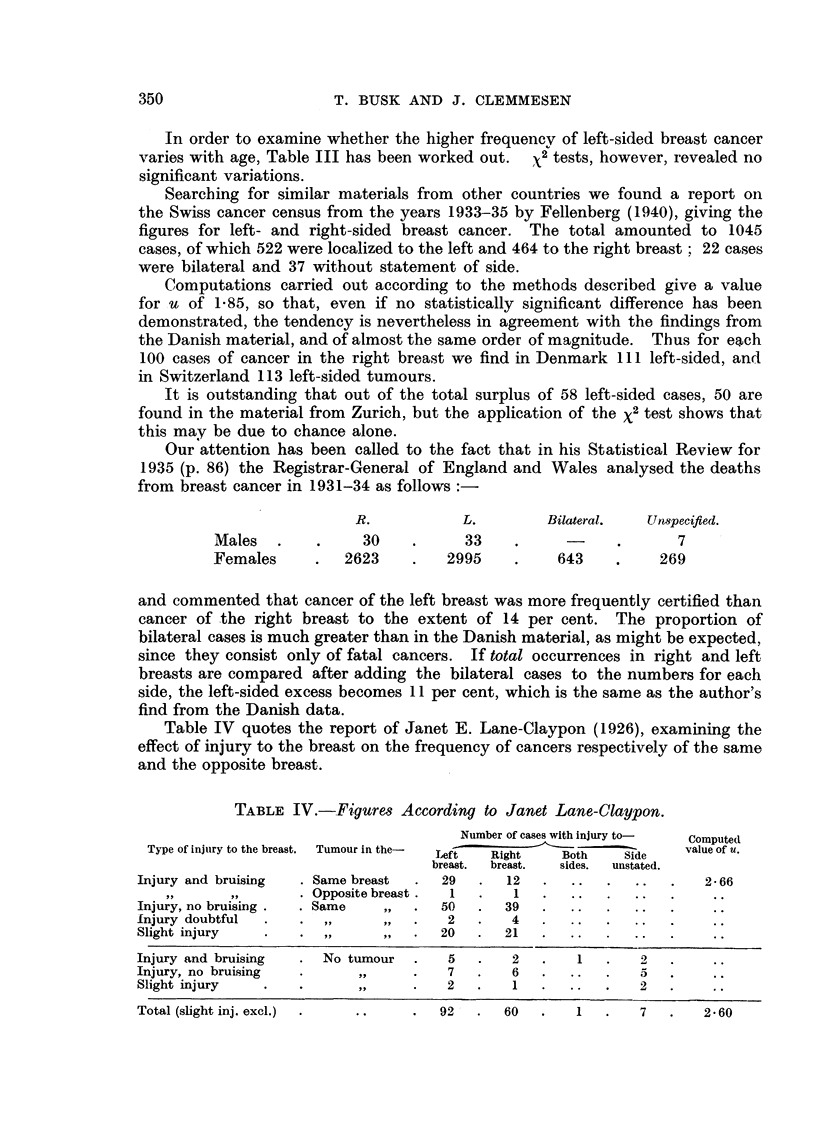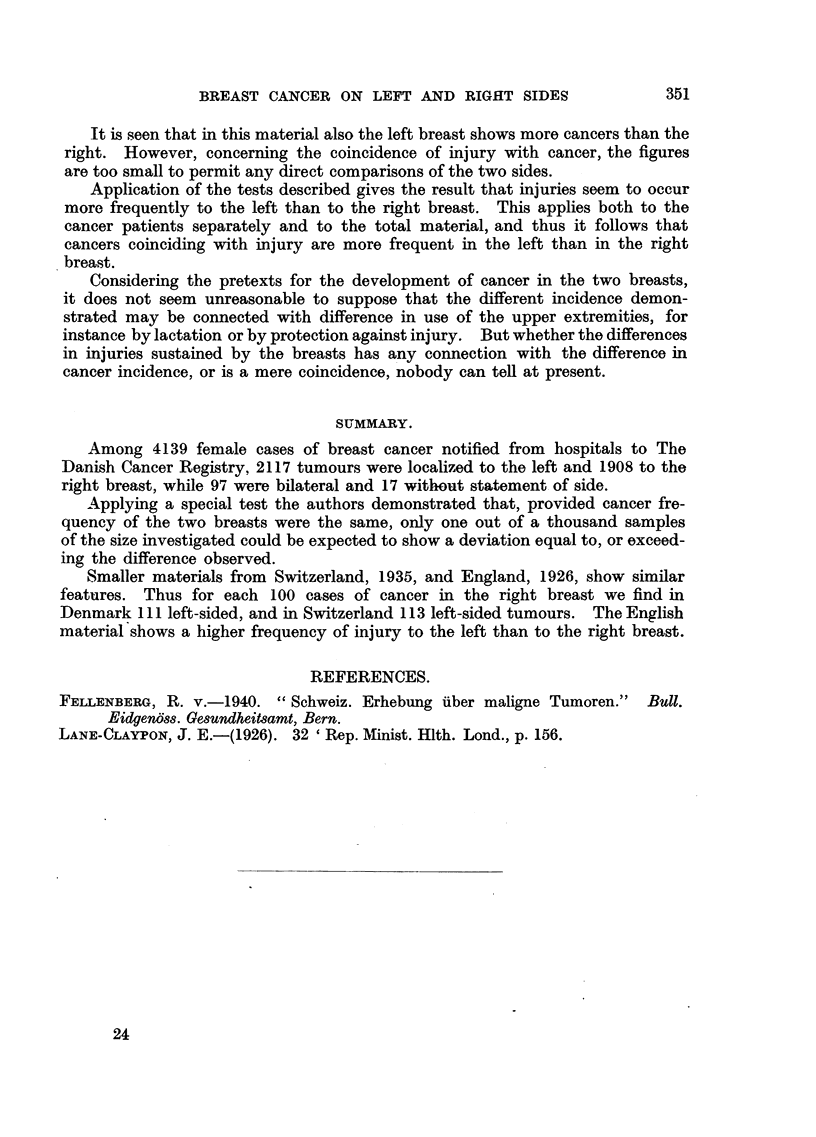# The Frequencies of Left- and Right-sided Breast Cancer

**DOI:** 10.1038/bjc.1947.31

**Published:** 1947-12

**Authors:** T. Busk, J. Clemmesen


### THE FREQUENCIES OF LEFT- AND RIGHT-SIDED BREAST CANCER.

T. BUSK AND J. CLEMMESEN.

*From the Danish Cancer Registry under The National Anti-Cancer League.*

Received for publication October 31, 1947.

BECAUSE of the anatomical and physiological symmetry of the breasts it is generally assumed that also their pathological symmetry goes without saying—nay even without documentation.

The question whether breast cancer arises with the same frequency in both breasts became topical to one of us while examining whether relatives with breast cancer were more likely than other persons to develop their tumours of that organ on the same side.

For our examination we have used the material of hospital cases notified to The Danish Cancer Registry. We are indebted to our medical colleagues for their interest, and the result that out of 4139 female cases of carcinoma from the years 1942–46 inclusive, only 17 have not been referred to any of the two sides.

The total Danish material will naturally also include cases from private practice known to the Cancer Registry only through death certificates. Because of the difficulties involved in obtaining accurate information as to the site of the tumour in many of these cases they have been left out of the present examination. They amount to about 130 cases each year. Thus 412 cases from the years 1942–44, known only from death certificates, consisted of 81 left-sided and 64 right-sided cases, together with 7 bilateral and 260 unstated cases.

Table I gives the number of cases for each of the two sides, distributed according to age. Similar tables showing the numbers of unmarried, married, divorced and widowed persons with breast cancer have also been worked out to examine if these conditions might influence the side of the tumour. The table gives these figures for the whole period, and for each year. The sum total shows 2117 tumours of the left and 1908 of the right breast. In 97 cases tumours were stated to have occurred on both sides, while the localization of 17 tumours was unknown.

The question now arises if this surplus of 209 cases in the left breast is statistically significant or may be due to chance.

In order to test the hypothesis that left- and right-sided breast cancers occur with equal frequency, the following considerations were made :

Consider $n$ observations of the same kind, by which the events $A_1, A_2, A_3$ occur with the probabilities $p_1, p_2, p_3$ respectively, where $p_1 + p_2 + p_3 = 1$. The probability that $A_1, A_2, A_3$ will occur $a_1, a_2, a_3$ times respectively is then according to the multinomial theorem :

$$p\left\{a_1, a_2, a_3\right\} = \frac{n!}{a_1!\, a_2!\, a_3!}\; p_1^{a_1}\, p_2^{a_2}\, p_3^{a_3}, \text{ where } a_1 + a_2 + a_3 = n.$$

If we put $p_1 = p_2 = p$ we get

$$p\left\{a_1, a_2, a_3\right\} = \frac{n!}{a_1!\, a_2!\, a_3!}\, p^{a_1 + a_2}\, (1 - 2p)^{a_3},$$

TABLE I.—*Cancer of Left and Right Breast.*

| Age. | 1942. L. | 1942. R. | 1942. Bilateral. | 1942. Unsp. | 1942. Total. | 1943. L. | 1943. R. | 1943. Bilateral. | 1943. Unsp. | 1943. Total. | 1944. L. | 1944. R. | 1944. Bilateral. | 1944. Unsp. | 1944. Total. |
|---|---|---|---|---|---|---|---|---|---|---|---|---|---|---|---|
| 15–19 | | | | | | | | | | | | | | | |
| 20–24 | 1 | 2 | | | 3 | | | | | | | | | | |
| 25–29 | | | 1 | | 1 | 2 | | | | 2 | 1 | 2 | | | 3 |
| 30–34 | 6 | 9 | 1 | | 16 | 7 | 8 | | | 15 | 16 | 10 | 2 | | 28 |
| 35–39 | 14 | 14 | 1 | | 29 | 21 | 18 | | 1 | 40 | 20 | 24 | | | 44 |
| 40–44 | 33 | 33 | 5 | | 71 | 40 | 42 | | 1 | 83 | 42 | 37 | 2 | | 81 |
| 45–49 | 49 | 39 | 6 | | 94 | 60 | 53 | 4 | | 117 | 55 | 50 | 3 | | 108 |
| 50–54 | 42 | 34 | 1 | | 77 | 32 | 45 | 4 | | 81 | 51 | 34 | 2 | | 87 |
| 55–59 | 63 | 44 | 4 | | 111 | 68 | 48 | 2 | | 118 | 60 | 44 | 3 | 2 | 109 |
| 60–64 | 49 | 44 | 4 | 1 | 98 | 65 | 60 | 2 | 1 | 128 | 63 | 43 | 2 | | 108 |
| 65–69 | 43 | 39 | 3 | | 85 | 50 | 37 | 7 | | 94 | 80 | 45 | 4 | | 129 |
| 70–74 | 29 | 28 | 2 | | 59 | 35 | 37 | 1 | | 73 | 40 | 33 | 3 | | 76 |
| 75–79 | 11 | 14 | 1 | | 26 | 22 | 20 | 3 | 1 | 46 | 20 | 20 | 2 | | 42 |
| 80–84 | 5 | 5 | | | 10 | 9 | 10 | 2 | 1 | 22 | 11 | 9 | | | 20 |
| 85–89 | 3 | 2 | | | 5 | 1 | 1 | 1 | | 3 | 4 | 5 | | | 9 |
| 90–94 | | | | | | | | | | | | | | | 2 |
| unstated | | | | | | | | | | | | | | 1 | 1 |
| Total | 348 | 307 | 29 | 1 | 685 | 412 | 379 | 26 | 5 | 822 | 464 | 357 | 23 | 3 | 847 |
| Of which unmarried | 65 | 57 | 6 | 1 | 129 | 82 | 73 | 7 | | 162 | 95 | 77 | 12 | | 184 |

| Age. | 1945. L. | 1945. R. | 1945. Bilateral. | 1945. Unsp. | 1945. Total. | 1946. L. | 1946. R. | 1946. Bilateral. | 1946. Unsp. | 1946. Total. | 1942–46. L. | 1942–46. R. | 1942–46. Bilateral. | 1942–46. Unsp. | 1942–46. Total. |
|---|---|---|---|---|---|---|---|---|---|---|---|---|---|---|---|
| 15–19 | | | | | | | 2 | | | 2 | | 2 | | | 2 |
| 20–24 | | 1 | | | 1 | 1 | | | | 1 | 2 | 3 | | | 5 |
| 25–29 | 4 | 2 | | | 6 | 4 | 1 | | | 5 | 11 | 5 | 1 | | 17 |
| 30–34 | 13 | 13 | | | 26 | 7 | 7 | | | 14 | 49 | 47 | 3 | | 99 |
| 35–39 | 21 | 20 | | | 41 | 32 | 28 | | | 60 | 108 | 104 | 1 | 1 | 214 |
| 40–44 | 49 | 34 | | | 83 | 46 | 40 | | | 86 | 210 | 186 | 7 | 1 | 404 |
| 45–49 | 52 | 44 | 2 | | 98 | 65 | 60 | 2 | 1 | 128 | 281 | 246 | 17 | 1 | 545 |
| 50–54 | 59 | 62 | 1 | | 122 | 56 | 56 | | | 112 | 240 | 231 | 8 | | 479 |
| 55–59 | 63 | 52 | 3 | 1 | 119 | 59 | 60 | | | 119 | 313 | 248 | 12 | 3 | 576 |
| 60–64 | 55 | 69 | | | 124 | 58 | 51 | 2 | | 111 | 290 | 267 | 10 | 2 | 569 |
| 65–69 | 51 | 63 | 1 | 2 | 117 | 53 | 43 | 6 | 1 | 103 | 277 | 227 | 21 | 3 | 528 |
| 70–74 | 36 | 47 | | 2 | 85 | 29 | 37 | | | 66 | 169 | 182 | 6 | 2 | 359 |
| 75–79 | 15 | 19 | | | 34 | 28 | 26 | | | 54 | 96 | 99 | 6 | 1 | 202 |
| 80–84 | 10 | 14 | | | 24 | 12 | 8 | 2 | 1 | 23 | 47 | 46 | 4 | 2 | 99 |
| 85–89 | 6 | 2 | | | 8 | 8 | 4 | | | 12 | 22 | 14 | 1 | | 37 |
| 90–94 | 1 | | | | 1 | | | | | | 2 | 1 | | | 3 |
| unstated | | | | | | | | | | | | | | 1 | 1 |
| Total | 435 | 442 | 7 | 5 | 889 | 458 | 423 | 12 | 3 | 896 | 2117 | 1908 | 97 | 17 | 4139 |
| Of which unmarried | 86 | 94 | 1 | | 181 | 92 | 96 | 2 | 1 | 191 | | | | | |

TABLE I—*continued.*

| | Unmarried women 1942–46. | | | | |
|---|---|---|---|---|---|
| | L. | R. | Both | Unsp. | Total. |
| 15–19 | | 2 | | | 2 |
| 20–24 | 2 | | | | 2 |
| 25–29 | 1 | 1 | | | 2 |
| 30–34 | 10 | 6 | 2 | | 18 |
| 35–39 | 19 | 13 | | | 32 |
| 40–44 | 51 | 50 | 2 | | 103 |
| 45–49 | 50 | 65 | 4 | | 119 |
| 50–54 | 37 | 49 | 4 | | 90 |
| 55–59 | 61 | 56 | 3 | | 120 |
| 60–64 | 63 | 54 | 3 | 1 | 121 |
| 65–69 | 52 | 47 | 4 | | 103 |
| 70–74 | 39 | 33 | 3 | | 75 |
| 75–79 | 18 | 15 | 2 | | 35 |
| 80–84 | 13 | 5 | | 1 | 19 |
| 85–89 | 4 | 1 | 1 | | 6 |
| 90–94 | | | | | |
| unstated | | | | | |
| Total | 420 | 397 | 28 | 2 | 847 |

and by the binomial theorem we get for the probability of $(a_1 + a_2)$ events of either of the two kinds $A_1$ and $A_2$ in $n$ observations :

$$p\left\{a_1 + a_2\right\} = \frac{n!}{(a_1 + a_2)!\, a_3!} (2p)^{a_1 + a_2} (1 - 2p)^{a_3}.$$

The probability that $A_1$ occurs just $a_1$ times in a series of $n$ observations where just $(a_1 + a_2)$ of the results belonged to either of the groups $A_1$ and and $A_2$ is then

$$p\left\{a_1, a_2, a_3 \,\middle|\, a_1 + a_2\right\} = \frac{(a_1 + a_2)!}{a_1!\, a_2!} (\tfrac{1}{2})^{a_1 + a_2} \quad (1)$$

This result is identical with the one obtained if $n_1$ observations are made with the probabilities $p_1 = \frac{1}{2}$ of the event $A_1$ and $p_2 = \frac{1}{2}$ of $A_2$ and the probability of getting the event $A_1$ just $a_1$ times is sought :

$$p\left\{a_1\right\} = \binom{n_1}{a_1}\left(\frac{1}{2}\right)^{n_1}$$

as we get the right side of (1) if we put $n_1 = a_1 + a_2 = n - a_3$.

The procedure involved in (1) : by $n$ observations $(n = a_1 + a_2 + a_3)$ to keep $(a_1 + a_2)$ at a certain fixed value is then tantamount to omitting the $a_3$ cases of the event $A_3$ and confining the considerations to the remaining $n - a_3$ observations in testing the hypothesis that $A_1$ and $A_2$ are equally frequent.

As an approximative test it can be asserted that the quantity

$$u = \frac{\frac{a_1}{n_1} - \frac{1}{2}}{\sqrt{\frac{1}{2} \cdot \frac{1}{2} \cdot \frac{1}{n_1}}} = \frac{a_1 - a_2}{\sqrt{n_1}}$$

approximately should be normally distributed with mean zero and variance 1, if $p_1 = p_2$.

For the application to the problems in question we interpret :

$A_1$ = tumour in the left breast ;
$A_2$ = tumour in the right breast ;
$A_3$ = bilateral or unstated localization of breast cancer ; and calculations then give $u = 3{\cdot}29$, corresponding to a value $P$ of about 1 per thousand.

Assuming that the left and right breast cancer was equally frequent, calculations according to the tests described have shown that only one out of a thousand samples of the size of the present data could be expected to show a deviation equal to, or exceeding the differences observed. Thus the preponderance of left-sided breast cancer can be considered as statistically significant, and according to the present material each hundred cases of cancer in the right breast will correspond to 111 of the left.

In working out the material we found some variations in the left-side preponderance of the different years. Especially the year 1945 showed a slightly higher frequency of right-sided tumours, and computations were therefore carried out to ascertain whether these variations would contradict the assumption of a constant inequality with regard to lateral distribution of breast cancers in all five years.

TABLE II.—*Number of Left-sided Breast Cancers per Thousand Cases with Known Localization.*

| Year. | Married women, etc. | Unmarried women. | Total. |
|---|---|---|---|
| 1942 | 531 | 533 | 531 |
| 1943 | 519 | 529 | 521 |
| 1944 | 569 | 552 | 565 |
| 1945 | 501 | 478 | 496 |
| 1946 | 528 | 489 | 520 |
| 1942–46 | 529 | 514 | 526 |
| $\chi^2$ | 6·59 | 2·73 | 8·50 |
| $f$ | 4 | 4 | 4 |
| $P$ | >0·10 | >0·50 | >0·05 |

The $\chi^2$ test carried out in Table II gives the value 8·50 with four degrees of freedom, and as this corresponds to $P >$ 5 per cent, it means that the material does not disprove the assumption of constant inequality in lateral distribution.

Considering the well-known differences in frequency of breast cancer among married and unmarried women, we have tested the corresponding values for breast cancer of each side for two groups, respectively of women not previously married, and of the remaining group of married, divorced and widowed women. We found for each hundred right-sided cancers, 105·8 left-sided tumours among unmarried, and 112·3 among the remaining women, and this difference between the two groups cannot be considered as significant.

TABLE III.—*Number of Left-sided Breast Cancers per Thousand Cases with Known Localization.*

| Age. | Married, divorced and widowed women. 1942. | 1943. | 1944. | 1945. | 1946. | 1942–46. | Unmarried women 1942–46. | Total 1942–46. |
|---|---|---|---|---|---|---|---|---|
| 34 | 333 | 539 | 591 | 483 | 556 | 505 | 591 | 521 |
| 35–39 | 462 | 514 | 436 | 486 | 556 | 494 | 594 | 509 |
| 40–44 | 480 | 500 | 517 | 623 | 567 | 539 | 505 | 530 |
| 45–49 | 580 | 548 | 566 | 559 | 556 | 561 | 435 | 533 |
| 50–54 | 574 | 438 | 614 | 505 | 517 | 527 | 430 | 510 |
| 55–59 | 609 | 554 | 610 | 574 | 500 | 568 | 521 | 558 |
| 60–64 | 506 | 510 | 588 | 455 | 537 | 516 | 538 | 521 |
| 65–69 | 533 | 586 | 660 | 449 | 530 | 556 | 525 | 550 |
| 70–74 | 468 | 466 | 542 | 406 | 451 | 466 | 542 | 482 |
| 75–79 | 476 | 525 | 433 | 440 | 500 | 482 | 545 | 492 |
| 80– | 538 | 500 | 423 | 464 | 583 | 495 | 739 | 538 |
| All ages | 531 | 519 | 569 | 501 | 528 | 529 | 514 | 526 |
| $\chi^2$ | 7·91 | 4·53 | 12·94 | 11·25 | 2·88 | 13·38 | 12·05 | 9·69 |
| $f$ | 10 | 10 | 10 | 10 | 10 | 10 | 10 | 10 |
| $P$ | >0·50 | >0·90 | >0·20 | >0·30 | >0·98 | >0·20 | >0·20 | >0·50 |

In order to examine whether the higher frequency of left-sided breast cancer varies with age, Table III has been worked out. $\chi^2$ tests, however, revealed no significant variations.

Searching for similar materials from other countries we found a report on the Swiss cancer census from the years 1933–35 by Fellenberg (1940), giving the figures for left- and right-sided breast cancer. The total amounted to 1045 cases, of which 522 were localized to the left and 464 to the right breast ; 22 cases were bilateral and 37 without statement of side.

Computations carried out according to the methods described give a value for $u$ of 1·85, so that, even if no statistically significant difference has been demonstrated, the tendency is nevertheless in agreement with the findings from the Danish material, and of almost the same order of magnitude. Thus for each 100 cases of cancer in the right breast we find in Denmark 111 left-sided, and in Switzerland 113 left-sided tumours.

It is outstanding that out of the total surplus of 58 left-sided cases, 50 are found in the material from Zurich, but the application of the $\chi^2$ test shows that this may be due to chance alone.

Our attention has been called to the fact that in his Statistical Review for 1935 (p. 86) the Registrar-General of England and Wales analysed the deaths from breast cancer in 1931–34 as follows :—

| | *R.* | *L.* | *Bilateral.* | *Unspecified.* |
|---|---|---|---|---|
| Males | 30 | 33 | — | 7 |
| Females | 2623 | 2995 | 643 | 269 |

and commented that cancer of the left breast was more frequently certified than cancer of the right breast to the extent of 14 per cent. The proportion of bilateral cases is much greater than in the Danish material, as might be expected, since they consist only of fatal cancers. If *total* occurrences in right and left breasts are compared after adding the bilateral cases to the numbers for each side, the left-sided excess becomes 11 per cent, which is the same as the author's find from the Danish data.

Table IV quotes the report of Janet E. Lane-Claypon (1926), examining the effect of injury to the breast on the frequency of cancers respectively of the same and the opposite breast.

TABLE IV.—*Figures According to Janet Lane-Claypon.*

| Type of injury to the breast. | Tumour in the— | Number of cases with injury to— Left breast. | Right breast. | Both sides. | Side unstated. | Computed value of $u$. |
|---|---|---|---|---|---|---|
| Injury and bruising | Same breast | 29 | 12 | .. | .. | 2·66 |
| ,, ,, | Opposite breast | 1 | 1 | .. | .. | .. |
| Injury, no bruising | Same ,, | 50 | 39 | .. | .. | .. |
| Injury doubtful | ,, ,, | 2 | 4 | .. | .. | .. |
| Slight injury | ,, ,, | 20 | 21 | .. | .. | .. |
| Injury and bruising | No tumour | 5 | 2 | 1 | 2 | .. |
| Injury, no bruising | ,, | 7 | 6 | .. | 5 | .. |
| Slight injury | ,, | 2 | 1 | .. | 2 | .. |
| Total (slight inj. excl.) | .. | 92 | 60 | 1 | 7 | 2·60 |

It is seen that in this material also the left breast shows more cancers than the right. However, concerning the coincidence of injury with cancer, the figures are too small to permit any direct comparisons of the two sides.

Application of the tests described gives the result that injuries seem to occur more frequently to the left than to the right breast. This applies both to the cancer patients separately and to the total material, and thus it follows that cancers coinciding with injury are more frequent in the left than in the right breast.

Considering the pretexts for the development of cancer in the two breasts, it does not seem unreasonable to suppose that the different incidence demonstrated may be connected with difference in use of the upper extremities, for instance by lactation or by protection against injury. But whether the differences in injuries sustained by the breasts has any connection with the difference in cancer incidence, or is a mere coincidence, nobody can tell at present.

### SUMMARY.

Among 4139 female cases of breast cancer notified from hospitals to The Danish Cancer Registry, 2117 tumours were localized to the left and 1908 to the right breast, while 97 were bilateral and 17 without statement of side.

Applying a special test the authors demonstrated that, provided cancer frequency of the two breasts were the same, only one out of a thousand samples of the size investigated could be expected to show a deviation equal to, or exceeding the difference observed.

Smaller materials from Switzerland, 1935, and England, 1926, show similar features. Thus for each 100 cases of cancer in the right breast we find in Denmark 111 left-sided, and in Switzerland 113 left-sided tumours. The English material shows a higher frequency of injury to the left than to the right breast.

### REFERENCES.

FELLENBERG, R. v.—1940. " Schweiz. Erhebung über maligne Tumoren." *Bull. Eidgenöss. Gesundheitsamt, Bern.*

LANE-CLAYPON, J. E.—(1926). 32 ' Rep. Minist. Hlth. Lond., p. 156.